# Synthesis, Structure, and Analgesic Properties of Halogen-Substituted 4-Hydroxy-2,2-dioxo-1*H*-2λ^6^,1-benzothiazine-3-carboxanilides

**DOI:** 10.3390/scipharm84030523

**Published:** 2016-06-17

**Authors:** Igor V. Ukrainets, Lidiya A. Petrushova, Svitlana V. Shishkina, Lyudmila V. Sidorenko, Galina Sim, Olga V. Kryvanych

**Affiliations:** 1 Department of Pharmaceutical Chemistry, National University of Pharmacy, 53 Pushkinska St., Kharkiv 61002, Ukraine; dika-l1@ya.ru (L.A.P.); slv.ludmila@i.ua (L.V.S.); oluska87@mail.ru (O.V.K.); 2 STC “Institute for Single Crystals”, National Academy of Sciences of Ukraine, 60 Nauki Ave., Kharkiv 61001, Ukraine; sveta@xray.isc.kharkov.com; 3 Department of Inorganic Chemistry, V. N. Karazin Kharkiv National University, 4 Svobody Sq., Kharkiv 61077, Ukraine; 4 Department of Pharmaceutical Chemistry, Far Eastern State Medical University, 35 Murav’eva-Amurskogo St., Khabarovsk 680000, Russia; sim.hab@mail.ru

**Keywords:** amidation, analgesia, anilide, synthesis, pain syndrome, 4-hydroxy-2,2-dioxo-1*H*-2λ^6^,1-benzothiazine-3-carboxamides, 2,1-benzothiazines

## Abstract

As potential new analgesics, the corresponding 4-hydroxy-2,2-dioxo-1*H*-2λ^6^,1-benzothiazine-3-carboxanilides have been obtained by amidation of ethyl 4-hydroxy-2,2-dioxo-1*H*-2λ^6^,1-benzothiazine-3-carboxylate with aniline and its halogenated analogsin boiling dry xylene. The peculiarities of the mass and nuclear magnetic resonance (^1^Н and ^13^С) spectra of the synthesized compounds are discussed. Using X-ray diffraction analysis, the ability of the compounds to form stable solvates with *N*,*N*-dimethylformamide has been shown on the example of 4-bromo-substituted derivative. It should be further studied to be considered in their crystallization. According to the results of the pharmacological testing conducted on the model of the thermal tail-flick (tail immersion test) among halogen-substituted 4-hydroxy-2,2-dioxo-1*H*-2λ^6^,1-benzothiazine-3-carboxanilides, substances which are considerably superior to meloxicam and piroxicam by their analgesic activity have been found. They are of interest for further profound studies.

## 1. Introduction

The search for promising, new substances with analgesic activity and the subsequent creation of highly effective and safe painkillers on this basis are among the critical tasks of pharmaceutical chemistry. It must be emphasized that in terms of modern ideas, such drugs should not cause addiction in human sand moreover, physical or psychological dependence, i.e., agonism with opioid receptors, is not acceptable in the mechanism of their analgesic action. Recently, with the purpose of searching for analgesics meeting such requirements, the agonists of neuronal nicotinic acetylcholine receptors (*n*AChR) were actively studied [1,2,3,4,5,6]. Furthermore, they are of interest as potential agents to fight the manifestations of age-related neurodegeneration (Alzheimer’s disease [7,8], Parkinson’s disease [9], and various types of dementia [10]).

**Figure 1:**
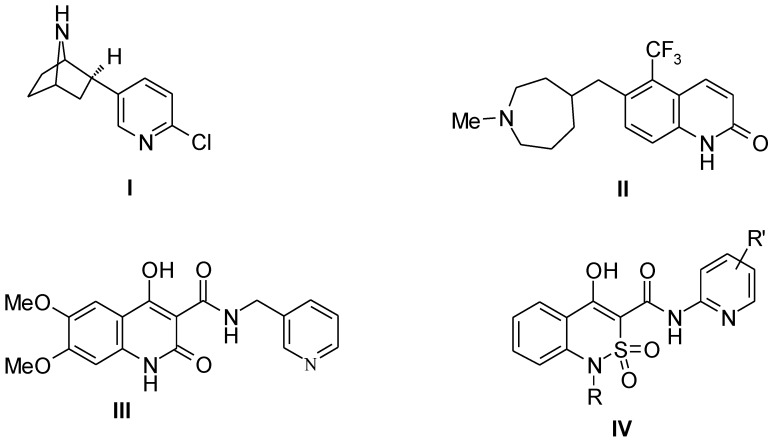
Natural (**I**) and synthetic (**II** and **III**) analgesics—agonists of nicotinic acetylcholine receptors [12,13,14] and their possible analogs (**IV**) by their mechanism of action.

The prime cause of the rapid development in this direction was the study of the structure and biological properties of the natural alkaloid epibatidine (**I**, Figure 1) with the chlorine-substituted pyridine core in its base [11]. In experiments in animals, this compound, isolated from the skin of the Ecuadorian tree frog *Epipedobates tricolor*, appeared to be 200–500 times more active than morphine; moreover, the resulting analgesia was not eliminated by the antagonists of opioid receptors (naloxone or naltrexone). In further research, it was found that the potent analgesic effect of epibatidine was through the activation of neuronal nicotinic acetylcholine receptors; after this discovery a lot of researchers began to pay particularly close attention to the representatives of this group of pharmacologically active substances [12].

Synthetic compounds of various chemical classes were studied [12]. However, the most successful findings were made, in the first place, among the derivatives of nitrogen-containing heterocycles. In particular, 5-(trifluoromethyl)-6-(1-methylazepan-4-yl)methyl-1*H*-quinolin-2-one (**II**) [13] and *N*-(3-pyridylmethyl)-4-hydroxy-6,7-dimethoxy-2-oxo-1,2-dihydroquinoline-3-carboxamide (**III**) appeared to be promising analgesics [14]. Their structural similarity with 1-R-4-hydroxy-*N*-(pyridin-2-yl)-2,2-dioxo-1*H*-2λ^6^,1-benzothiazine-3-carboxamides (**IV**) [15,16] allowed us to assume with high probability that derivatives of 2,1-benzothiazine would also be agonists of nicotinic acetylcholine receptors by the mechanism of the analgesic action. It is of interest that *N*-(5-chloropyridin-2-yl)amide (**IV**, R = Me, R′ = 5-Cl) showed the most potent analgesic properties among the substances of this series [15]. This fact was the impetus for involvement of halogen-substituted 4-hydroxy-2,2-dioxo-1*H*-2λ^6^,1-benzothiazine-3-carboxanilides in our expanded search for new analgesics.

**Figure 2:**
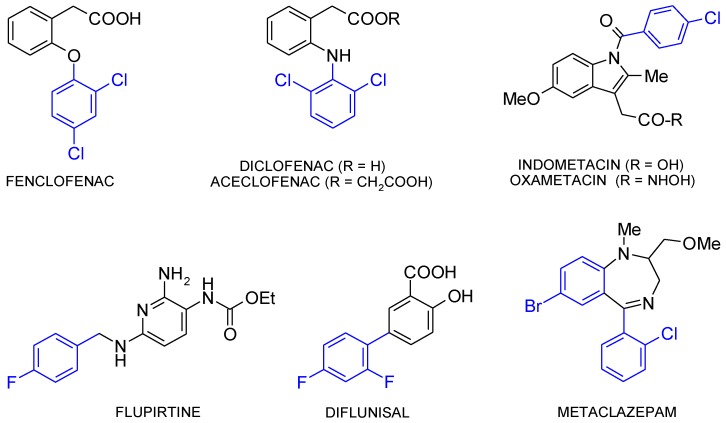
Analgesics containing the fragments of halogen-substituted benzene [18].

Several important points were considered when choosing exactly these compounds as the study objects. First, replacement of pyridylamine fragments in compounds **IV** by anilide ones can be considered as one of the most obvious variants of optimization of the base molecule according to the methodology of “bioisosteric replacements” [17]. Secondly, halogen-substituted benzene cores have a positive effect on the analgesic properties of compounds of different chemical classes as evidenced by their presence in the structure of many drugs of this pharmacological group (Figure 2) [18]. Finally, the high analgesic activity is characteristic for 4-hydroxy-2,2-dioxo-1*H*-2λ^6^,1-benzothiazine-3-carboxamides with different substituents in the terminal amide fragment, including hetaryl [15,16,19,20], aryl alkyl amide [21], and anilide [22,23,24,25] ones.

## 2. Materials and Methods

### 2.1. Chemistry

^1^Н- and ^13^С-NMR spectra were acquired on a Varian Mercury-400 (Varian Inc., Palo Alto, CA, USA) instrument (400 and 100 MHz, respectively) in DMSO-*d*_6_ with tetramethylsilane as internal standard. The chemical shift values were recorded on a *δ* scale and the coupling constants (*J*) in hertz. The following abbreviations were used in reporting spectra: s = singlet, d = doublet, t = triplet, m = multiplet. The electron impact mass spectra were recorded on a Varian 1200 L (Varian Inc., Walnut Creek, CA, USA) mass spectrometer with complete scanning in the *m*/*z* range from 35 to 700 and direct sample inlet. The electron impact ionization was at 70 eV. Elemental analysis was performed on a Euro Vector EA-3000 (Eurovector SPA, Redavalle, Italy) microanalyzer. Melting points were determined in a capillary using a Stuart SMP10 (Bibby Scientific Limited, Stone, UK) digital melting point apparatus.

### 2.2. General Procedure for the Synthesis of *N*-aryl-4-hydroxy-2,2-dioxo-1*H*-2λ^6^,1-benzothiazine-3-carboxamides (***2a**–**l***)

A mixture of ethyl ester **1** (2.69 g, 0.01 mol), corresponding aniline (0.01 mol), and dry xylene (5 mL) was kept for 1 h at 150 °С on a liquid metal bath using a suitable air-cooled distilling column that allowed us to distill off the ethanol formed without removing the xylene solvent. The reaction mixture was cooled, EtOH (5 mL) was added, and the mixture was left for several hours at room temperature. The crystalline amide **2** precipitate was filtered off, washed with cold EtOH, dried, and recrystallized from the suitable solvent. Anilides **2a**–**l** were colorless or white with yellowish tinted crystals.

*4-Hydroxy-*N*-phenyl-2,2-dioxo-1*Н*-2λ^6^,1-benzothiazine-3-carboxamide* (**2a**). Yield: 91%; melting point (mp) 207–209 °C (methanol); ^1^H-NMR (400 MHz, DMSO-*d*_6_): δ 15.40 (br. s, 1H, 4-ОН), 12.25 (br. s, 1H, SO_2_NH), 9.47 (s, 1Н, CONH), 8.00 (d, 1Н, *J* = 8.0 Hz, Н-5), 7.67–7.56 (m, 3Н, Н-7,2′,6′), 7.39 (t, 2Н, *J* = 7.7 Hz, Н-3′,5′), 7.27 (t, 1Н, *J* = 7.7 Hz, Н-6), 7.23–7.15 (m, 2Н, H-8,4′). ^13^C-NMR (100 MHz, DMSO-*d*_6_): δ167.8 (С-ОН), 163.7 (С=О), 136.6 (C-1′), 134.3 (С-8а), 128.1 (С-7), 126.2 (C-3′,5′), 124.5 (C-4′), 123.4 (С-4a), 122.9 (С-5), 121.2 (C-2′,6′), 119.1 (С-6), 118.2 (С-8), 104.5 (C-3). Mass Spectrum (MS) (*m*/*z*, %): 316 [M]^+^ (3.7), 224 (1.0), 93 (100), 92 (12.7), 77 (10.6). Analytical Calculated (Anal. Calcd.) for C_15_H_12_N_2_O_4_S: C, 56.95; H, 3.82; N, 8.86; S 10.14%. Found: C, 57.03; H, 3.89; N, 8.78; S 10.21%.

N*-(2-Fluorophenyl)-4-hydroxy-2,2-dioxo-1*Н*-2λ^6^,1-benzothiazine-3-carboxamide* (**2b**). Yield: 86%; mp 212–214 °C (methanol–methylene chloride, 1:1); ^1^H-NMR (400 MHz, DMSO-*d*_6_): δ 15.26 (br. s, 1H, 4-OH), 12.31 (br. s, 1H, SO_2_NH), 9.72 (s, 1H, CONH), 8.20 (t, 1Н, *J* = 7.8 Hz, Н-3′), 8.00 (d, 1H, *J* = 8.0 Hz, H-5), 7.64 (t, 1Н, *J* = 7.7 Hz, Н-7), 7.28 (t, 1Н, *J* = 7.6 Hz, Н-6), 7.24–7.18 (m, 4Н, Н-8,4′,5′,6′). ^13^C-NMR (100 MHz, DMSO-*d*_6_): δ168.9 (С-ОН), 164.3 (С=О), 154.1/152.0 (d, ^1^*J*_C__–__F_ = 243 Hz, C-2′-F), 137.8 (С-8а), 136.3 (С-7), 124.5 (C-5′), 126.3 (C-4′), 126.1/126.0 (d, ^2^*J*_C-F_ = 14.8 Hz, C-1′), 124.6 (С-6′), 124.5 (С-5), 123.6 (C-4a), 123.1 (С-6), 118.2 (С-8), 115.5/115.3 (d, ^2^*J*_C-F_ = 19.3 Hz, C-3′), 103.8 (C-3). MS (*m*/*z*, %): 334 [M]^+^ (1.4), 224 (1.2), 111 (100), 110 (11.2), 77 (12.5). Anal. Calcd. for C_15_H_11_FN_2_O_4_S: C, 53.89; H, 3.32; N, 8.38; S 9.59%. Found: C, 53.97; H, 3.38; N, 8.30; S 9.52%.

N*-(3-Fluorophenyl)-4-hydroxy-2,2-dioxo-1*Н*-2λ^6^,1-benzothiazine-3-carboxamide* (**2c**). Yield: 93%; mp 218–220 °C (methanol–methylene chloride, 1:1); ^1^H-NMR (400 MHz, DMSO-*d*_6_): δ 15.30 (br. s, 1H, 4-OH), 12.32 (br. s, 1H, SO_2_NH), 9.61 (s, 1H, CONH), 8.00 (d, 1H, *J* = 8.0 Hz, H-5), 7.66–7.58 (m, 2H, H-7,2′), 7.42–7.25 (m, 3H, H-6,4′,6′), 7.21 (d, 1Н, *J* = 8.2 Hz, Н-8), 6.94 (t, 1Н, *J* = 7.8 Hz, Н-5′). ^13^C-NMR (100 MHz, DMSO-*d*_6_): δ167.0 (С-ОН), 163.1/160.7 (d, ^1^*J*_C-F_ = 249 Hz, C-3′-F), 164.2 (С=О), 141.8 (C-1′), 136.7 (C-5′), 133.9 (С-8a), 130.5 (C-7), 123.4 (C-4a), 122.8 (С-5), 119.5 (С-6), 118.0 (С-6′), 116.8 (С-8), 115.1/115.0 (d, ^2^*J*_C-F_ = 16.2 Hz, C-2′), 111.2/111.0 (d, ^2^*J*_C-F_ = 21.7 Hz, C-4′), 105.3 (C-3). MS (*m*/*z*, %): 334 [M]^+^ (2.8), 224 (6.7), 132 (10.3), 111 (100), 104 (16.8), 92 (34.5), 77 (10.2). Anal. Calcd. for C_15_H_11_FN_2_O_4_S: C, 53.89; H, 3.32; N, 8.38; S 9.59%. Found: C, 53.95; H, 3.35; N, 8.42; S 9.65%.

N*-(4-Fluorophenyl)-4-hydroxy-2,2-dioxo-1*Н*-2λ^6^,1-benzothiazine-3-carboxamide* (**2d**). Yield: 95%; mp 235–237 °C (methanol–methylene chloride, 1:2); ^1^H-NMR (400 MHz, DMSO-*d*_6_): δ 15.25 (br. s, 1H, 4-OH), 12.24 (br. s, 1H, SO_2_NH), 9.47 (s, 1H, CONH), 7.99 (d, 1H, *J* = 8.0 Hz, H-5), 7.67–7.58 (m, 3H, H-7,2′,6′), 7.27 (t, 1Н, *J* = 7.7 Hz, Н-6), 7.21 (d, 1H, *J* = 8.2 Hz, H-8), 7.15 (t, 2H, *J* = 8.6 Hz, H-3′,5′). ^13^C-NMR (100 MHz, DMSO-*d*_6_): δ167.7 (С-ОН), 163.7 (С=О), 160.2/157.7 (d, ^1^*J*_C-F_ = 250 Hz, C-4′-F), 136.7 (С-8а), 134.2 (С-1′), 128.2 (C-7), 126.0 (C-2′,6′), 123.6 (C-5), 123.4 (C-4a), 121.1 (С-6), 119.5 (С-8), 115.6/115.4 (d, ^2^*J*_C-F_ = 24.2 Hz, C-3′,5′), 104.5 (C-3). MS (*m*/*z*, %): 334 [M]^+^ (6.3), 224 (8.6), 132 (8.0), 111 (100), 92 (13.9), 83 (17.1). Anal. Calcd. for C_15_H_11_FN_2_O_4_S: C, 53.89; H, 3.32; N, 8.38; S 9.59%. Found: C, 53.81; H, 3.26; N, 8.302; S 9.51%.

N*-(3,4-Difluorophenyl)-4-hydroxy-2,2-dioxo-1*Н*-2λ^6^,1-benzothiazine-3-carboxamide* (**2e**). Yield: 90%; mp 217–219 °C (methanol); ^1^H-NMR (400 MHz, DMSO-*d*_6_): δ15.27 (br. s, 1H, 4-ОН),12.23 (br. s, 1H, SO_2_NH), 9.57 (s, 1Н, CONH),7.99 (d, 1Н, *J* = 7.9 Hz, Н-5), 7.79 (d, 1Н, *J* = 7.8 Hz, Н-2′), 7.63 (t, 1Н, *J* = 7.7Hz, Н-7), 7.37–7.31 (m, 2Н, Н-5′,6′), 7.26 (t, 1Н, *J* = 7.5Hz, Н-6), 7.20 (d, 1Н, *J* = 8.2Hz, Н-8). ^13^C-NMR (100 MHz, DMSO-*d*_6_): δ 167.0 (С-ОН), 163.3 (С=О), 149.9/147.5 (d, ^1^*J*_C-F_ = 244 Hz, C-3′-F), 147.2/144.8 (d, ^1^*J*_C-F_ = 241 Hz, C-4′-F), 136.7 (С-8а), 134.0 (С-1′), 128.2 (C-7), 126.1 (C-6′), 123.4 (C-4a), 122.9/122.7 (d, ^2^*J*_C-F_ = 16.7 Hz, C-5′), 119.5 (C-5), 117.9 (C-6), 117.5 (C-8), 110.8/110.6 (d, ^2^*J*_C-F_ = 17.9 Hz, C-2′), 105.2 (C-3). MS (*m*/*z*, %): 352 [M]^+^ (13.0), 224 (1.7), 129 (100), 128 (20.4), 92 (12.0), 77 (57.1). Anal. Calcd. for C_15_H_10_F_2_N_2_O_4_S: C, 51.14; H, 2.86; N, 7.95; S 9.10%. Found: C, 51.21; H, 2.93; N, 7.90; S 9.17%.

N*-(2-Chlorophenyl)-4-hydroxy-2,2-dioxo-1*Н*-2λ^6^,1-benzothiazine-3-carboxamide* (**2f**). Yield: 85%; mp 223–225 °C (methanol–methylene chloride, 1:1); ^1^H-NMR (400 MHz, DMSO-*d*_6_): δ 15.23 (br. s, 1H, 4-OH), 12.28 (br. s, 1H, SO_2_NH), 9.88 (s, 1H, CONH), 8.27 (d, 1Н, *J* = 8.0 Hz, Н-3′), 8.01 (d, 1H, *J* = 8.0 Hz, H-5), 7.62 (t, 1Н, *J* = 7.6 Hz, Н-7), 7.50 (d, 1Н, *J* = 8.4 Hz, Н-6′), 7.36 (t, 1Н, *J* = 7.7 Hz, Н-5′), 7.28 (t, 1Н, *J* = 7.8 Hz, Н-6), 7.24–7.17 (m, 2Н, Н-8,4′). ^13^C-NMR (100 MHz, DMSO-*d*_6_): δ169.1 (С-ОН), 164.4 (С=О), 137.9 (C-1′), 136.2 (С-8а), 134.6 (C-3′), 133.3 (C-5′), 129.4 (С-7), 128.9 (C-2′-Cl), 127.7 (C-4′), 126.3 (C-6′), 123.7 (С-4a), 123.0 (С-5), 122.6 (С-6), 118.4 (С-8), 103.7 (C-3). MS (*m*/*z*, %): 350/352 [M]^+^ (2.3/1.7), 315 (7.2), 224 (1.9), 127/129 (100/25.2), 77 (10.1). Anal. Calcd. for C_15_H_11_ClN_2_O_4_S: C, 51.36; H, 3.16; N, 7.99; S 9.14%. Found: C, 51.44; H, 3.23; N, 8.07; S 9.06%.

N*-(3-Chlorophenyl)-4-hydroxy-2,2-dioxo-1*Н*-2λ^6^,1-benzothiazine-3-carboxamide* (**2g**). Yield: 89%; mp 204–206 °C (methanol–methylene chloride, 1:1); ^1^H-NMR (400 MHz, DMSO-*d*_6_): δ 15.30 (br. s, 1H, 4-OH), 12.33 (br. s, 1H, SO_2_NH), 9.59 (s, 1H, CONH), 8.00 (d, 1H, *J* = 7.9 Hz, H-5), 7.80 (s, 1Н, Н-2′), 7.63 (t, 1Н, *J* = 7.7 Hz, Н-7), 7.48 (d, 1Н, *J* = 7.4 Hz, Н-6′), 7.37 (t, 1Н, *J* = 7.7 Hz, Н-5′), 7.26 (t, 1Н, *J* = 7.5 Hz, Н-6), 7.22–7.17 (m, 2Н, Н-8,4′). ^13^C-NMR (100 MHz, DMSO-*d*_6_): δ167.6 (С-ОН), 163.6 (С=О), 138.7 (C-1′), 136.6 (С-8а), 133.8 (C-3′-Cl), 130.4 (C-5′), 128.1 (С-7), 126.2 (C-4′), 124.3 (C-2′), 123.4 (С-4a), 122.6 (С-5), 120.5 (C-6′), 119.4 (С-6), 117.8 (С-8), 105.3 (C-3). MS (*m*/*z*, %): 350/352 [M]^+^ (8.2/4.9), 224 (1.2), 127/129 (100/10.8), 77 (21.4). Anal. Calcd. for C_15_H_11_ClN_2_O_4_S: C, 51.36; H, 3.16; N, 7.99; S 9.14%. Found: C, 51.42; H, 3.11; N, 7.91; S 9.20%.

N*-(4-Chlorophenyl)-4-hydroxy-2,2-dioxo-1*Н*-2λ^6^,1-benzothiazine-3-carboxamide* (**2h**). Yield: 92%; mp 241–243 °C (methanol–methylene chloride, 1:2); ^1^H-NMR (400 MHz, DMSO-*d*_6_): δ 15.36 (br. s, 1H, 4-OH), 12.27 (br. s, 1H, SO_2_NH), 9.52 (s, 1H, CONH), 7.99 (d, 1H, *J* = 8.0 Hz, H-5), 7.66–7.57 (m, 3H, H-7,2′,6′), 7.38 (d, 2H, *J* = 8.4 Hz, H-3′,5′), 7.27 (t, 1Н, *J* = 7.7 Hz, Н-6), 7.21 (d, 1H, *J* = 8.2 Hz, H-8). ^13^C-NMR (100 MHz, DMSO-*d*_6_): δ168.3 (С-ОН), 163.7 (С=О), 137.9 (C-1′), 136.8 (С-8а), 133.5 (C-4′-Cl), 129.0 (C-3′,5′), 128.7 (С-7), 126.3 (C-2′,6′), 123.5 (C-4a), 122.3 (С-5), 120.9 (C-6), 119.5 (С-8), 105.0 (C-3). MS (*m*/*z*, %): 350/352 [M]^+^ (41.8/24.9), 224 (5.7), 127/129 (100/11.4), 125 (16.3), 105 (30.7), 104 (33.1), 103 (39.7), 92 (30.5), 77 (63.9), 76 (30.4). Anal. Calcd. for C_15_H_11_ClN_2_O_4_S: C, 51.36; H, 3.16; N, 7.99; S 9.14%. Found: C, 51.44; H, 3.23; N, 8.07; S 9.08%.

N*-(2,5-Dichlorophenyl)-4-hydroxy-2,2-dioxo-1*Н*-2λ^6^,1-benzothiazine-3-carboxamide* (**2i**). Yield: 86%; mp 235–237 °C (methanol–methylene chloride, 1:1); ^1^H-NMR (400 MHz, DMSO-*d*_6_): δ 15.32 (br. s, 1H, 4-OH), 12.36 (br. s, 1H, SO_2_NH), 10.02 (s, 1H, CONH), 8.39 (s, 1H, H-6′), 8.01 (d, 1H, *J* = 7.8 Hz, H-5), 7.64 (t, 1Н, *J* = 7.6 Hz, Н-7), 7.52 (d, 1Н, *J* = 8.4 Hz, Н-4′), 7.28 (t, 1Н, *J* = 7.6 Hz, Н-6), 7.24–7.19 (m, 2Н, Н-8,5′). ^13^C-NMR (100 MHz, DMSO-*d*_6_): δ167.8 (С-ОН), 164.1 (С=О), 136.2 (C-5′-Cl), 135.0 (C-1′), 134.3 (С-8а), 131.7 (C-3′), 130.5 (С-7), 130.0 (C-2′-Cl), 126.5 (C-4′), 125.2 (C-6′), 123.3 (С-4a), 122.4 (С-5), 121.6 (С-6), 118.1 (С-8), 104.6 (C-3). MS (*m*/*z*, %): 384/386/388 [M]^+^ (4.5/2.8/0.6), 350/352 (11.3/2.9), 224 (1.3), 161/163/165 (79.5/15.2/2.9),145 (10.0), 135 (21.3), 133 (25.1), 132 (25.8), 120 (28.2), 119 (42.4), 104 (58.1), 103 (44.4), 102 (28.3), 90 (58.3), 88 (28.4), 77 (100), 76 (63.4). Anal. Calcd. for C_15_H_10_Cl_2_N_2_O_4_S: C, 46.77; H, 2.62; N, 7.27; S 8.32%. Found: C, 46.86; H, 2.69; N, 7.19; S 8.26%.

N*-(2-Bromophenyl)-4-hydroxy-2,2-dioxo-1*Н*-2λ^6^,1-benzothiazine-3-carboxamide* (**2j**). Yield: 84%; mp 223–225 °C (methanol–methylene chloride, 1:2); ^1^H-NMR (400 MHz, DMSO-*d*_6_): δ 15.24 (br. s, 1H, 4-OH), 12.26 (br. s, 1H, SO_2_NH), 9.76 (s, 1H, CONH), 8.19 (d, 1Н, *J* = 8.4 Hz, Н-3′), 8.00 (d, 1H, *J* = 8.0 Hz, H-5), 7.67–7.58 (m, 2Н, Н-7,6′), 7.39 (t, 1Н, *J* = 7.6 Hz, Н-5′), 7.27 (t, 1Н, *J* = 7.6 Hz, Н-6), 7.21 (d, 1H, *J* = 8.0 Hz, H-8), 7.13 (t, 1H, *J* = 7.4 Hz, Н-4′). ^13^C-NMR (100 MHz, DMSO-*d*_6_): δ169.5 (С-ОН), 164.9 (С=О), 138.5 (C-1′), 136.1 (С-8а), 135.5 (C-3′), 132.6 (С-7), 128.1 (C-5′), 126.9 (C-6′), 126.3 (C-4′), 124.5 (С-4a), 123.1 (С-5), 120.4 (C-6), 118.5 (С-8), 115.9 (С-2′-Br), 103.5 (C-3). MS (*m*/*z*, %): 394/396 [M]^+^ (3.2/2.8), 315 (6.8), 224 (4.6), 197/199 (29.6/32.3), 171/173 (18.2/15.5), 120 (14.9), 119 (20.1), 92 (17.2), 90 (100), 82 (12.8), 81 (14.5). Anal. Calcd. for C_15_H_11_BrN_2_O_4_S: C, 45.58; H, 2.81; N, 7.09; S 8.11%. Found: C, 45.50; H, 2.74; N, 7.00; S 8.18%.

N*-(3-Bromophenyl)-4-hydroxy-2,2-dioxo-1*Н*-2λ^6^,1-benzothiazine-3-carboxamide* (**2k**). Yield: 90%; mp 212–214 °C (methanol–methylene chloride, 1:2); ^1^H-NMR (400 MHz, DMSO-*d*_6_): δ 15.27 (br. s, 1H, 4-OH), 12.21 (br. s, 1H, SO_2_NH), 9.56 (s, 1H, CONH), 8.00 (d, 1H, *J* = 8.0 Hz, H-5), 7.92 (s, 1Н, Н-2′), 7.62 (t, 1Н, *J* = 7.7 Hz, Н-7), 7.52 (d, 1Н, *J* = 7.3 Hz, Н-6′), 7.36–7.31 (m, 2Н, Н-5′, 4′), 7.27 (t, 1Н, *J* = 7.6 Hz, Н-6), 7.21 (d, 1Н, *J* = 8.2 Hz, Н-8). ^13^C-NMR (100 MHz, DMSO-*d*_6_): δ168.4 (С-ОН), 164.3 (С=О), 138.9 (C-1′), 136.7 (С-8а), 134.0 (C-5′), 130.9 (С-7), 128.1 (C-3′-Br), 126.2 (C-2′), 123.5 (C-4′), 122.8 (С-4a), 121.5 (С-6′), 119.9 (C-5), 119.5 (С-6), 118.0 (С-8), 105.4 (C-3). MS (*m*/*z*, %): 394/396 [M]^+^ (7.9/5.1), 224 (3.7), 197/199 (30.7/30.5), 171/173 (100/88.8), 119 (30.9), 104 (25.0), 92 (53.7), 90 (65.5), 82 (12.3), 81 (23.3), 77 (17.4). Anal. Calcd. for C_15_H_11_BrN_2_O_4_S: C, 45.58; H, 2.81; N, 7.09; S 8.11%. Found: C, 45.64; H, 2.75; N, 6.99; S 8.07%.

N*-(4-Bromophenyl)-4-hydroxy-2,2-dioxo-1*Н*-2λ^6^,1-benzothiazine-3-carboxamide* (**2l**). Yield: 95%; mp 229–231 °C (methanol–methylene chloride, 1:3); ^1^H-NMR (400 MHz, DMSO-*d*_6_): δ 15.29 (br. s, 1H, 4-OH), 12.26 (br. s, 1H, SO_2_NH), 9.52 (s, 1H, CONH), 7.99 (d, 1H, *J* = 8.0 Hz, H-5), 7.63 (t, 1Н, *J* = 7.7 Hz, Н-7), 7.57 (d, 2H, *J* = 8.5 Hz, H-2′,6′), 7.51 (d, 2H, *J* = 8.5 Hz, H-3′,5′), 7.26 (t, 1Н, *J* = 7.6 Hz, Н-6), 7.21 (d, 1H, *J* = 8.2 Hz, H-8). ^13^C-NMR (100 MHz, DMSO-*d*_6_): δ167.4 (С-ОН), 163.5 (С=О), 136.8 (C-1′), 134.1 (С-8а), 131.7 (C-3′,5′), 128.3 (С-7), 126.1 (C-2′,6′), 123.0 (C-4a), 121.2 (C-5′), 119.5 (С-6), 118.0 (С-8), 116.3 (C-4′-Br), 104.9 (C-3). MS (*m*/*z*, %): 394/396 [M]^+^ (1.5/1.7), 224 (5.4), 197/199 (1.7/1.5), 171/173 (84.5/84.0), 132 (12.2), 119 (8.8), 104 (44.2), 92 (100), 91 (28.5), 77 (11.4). Anal. Calcd. for C_15_H_11_BrN_2_O_4_S: C, 45.58; H, 2.81; N, 7.09; S 8.11%. Found: C, 45.65; H, 2.86; N, 7.13; S 8.17%.

### 2.3. X-ray Structural Analysis

Crystal data for *N*-(4-bromophenyl)-4-hydroxy-2,2-dioxo-1*Н*-2λ^6^,1-benzothiazine-3-carboxamide dimethylformamide monosolvate: C_15_H_11_BrN_2_O_4_S·C_3_H_7_NO, colorless, monoclinic (DMF, mp 197–199 °C decomposition). At 20 °C *a* = 21.592(4), *b* = 12.809(1), *c* = 29.564(5) Å, *β* = 134.04(3)°, *V* = 5878(2) Å^3^, *M*_r_ = 468.32, *Z* = 12, space group P2_1_/c, *d*_calc_ = 1.588 g/сm^3^, μ(MoK_α_) = 2.240 mm^−1^, F(000) = 2856. The unit cell parameters and intensities of 40460 reflections (10335 independent, *R*_int_ = 0.119) were measured on an Xcalibur-3 (Oxford Diffraction Limited, Oxford, UK) diffractometer (MoK_α_ radiation, ССD detector, graphite monochromator, ω-scanning to 2θ_max_ = 50°). The structure was solved by the direct method using the SHELXTL program package (Institute of Inorganic Chemistry, Göttingen, Germany) [26]. Absorption correction was made using a semi-empirical multi-scan method (*T*_min_ = 0.553, *T*_max_ = 0.807). The hydrogen atom positions were revealed by differential synthesis of electron density and refined according to the “rider” model with *U*_iso_ = *nU*_eq_ for the non-hydrogen atom bonded to a given hydrogen atom (*n* = 1.5 for methyl group, *n* = 1.2 for the rest of the hydrogen atoms). The hydroxyl and amino group hydrogen atoms participating in hydrogen bonds were refined in isotropic approximation. The structure was refined using *F*^2^ full-matrix least-squares analysis in the anisotropic approximation for non-hydrogen atoms to *wR*_2_ = 0.173 for 10293 reflections (*R*_1_ = 0.069 for 799 reflections with *F* > 4σ (*F*), *S* = 0.902). CCDC 1474415 contains the supplementary crystallographic data for this paper. These data can be obtained free of charge from the Cambridge Crystallographic Data Centre [27].

### 2.4. Pharmacology

All biological experiments were carried out in full accord with the European Convention on the Protection of Vertebrate Animals Used for Experimental and Other Scientific Purposes and the Ukrainian Law No. 3447-IV ”On protection of animals from severe treatment” (2006) (project ID 3410U14 approved October 15, 2015). The analgesic activities of the synthesized 4-hydroxy-2,2-dioxo-1*H*-2λ^6^,1-benzothiazine-3-carboxanilides **2а**–**l** were studied compared to piroxicam (Jenapharm, Jena, Germany) and meloxicam (Boehringer Ingelheim, Ingelheim am Rhein, Germany), two structurally similar compounds, on the model of the thermal tail-flick procedure in white rats (tail immersion test) [28]. The conditions of our pharmacological experiments were previously described in detail [21]. The test compounds and the reference drugs were administered orally in a screening dose of 20 mg/kg.

## 3. Results and Discussion

### 3.1. Chemistry

**Scheme 1:**
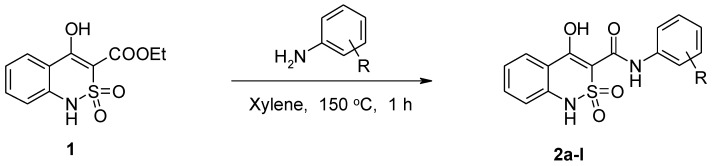
Synthesis of 4-hydroxy-2,2-dioxo-1*H*-2λ^6^,1-benzothiazine-3-carboxanilides **2a**–**l**. **2**: **а**R = H; **b**R = 2-F; **c**R = 3-F; **d**R = 4-F; **e**R = 3,4-F_2_; **f**R = 2-Cl; **g**R = 3-Cl; **h**R = 4-Cl; **i**R = 2,5-Cl_2_; **j**R = 2-Br; **k**R = 3-Br; **l**R = 4-Br.

The synthesis of halogen-substituted 4-hydroxy-2,2-dioxo-1*H*-2λ^6^,1-benzothiazine-3-carboxanilides **2а**–**l** was carried out by amidation of ethyl 4-hydroxy-2,2-dioxo-1*H*-2λ^6^,1-benzothiazine-3-carboxylate (**1**) previously described [29] with the corresponding anilines in boiling dry xylene (Scheme 1). This rather simple method allows for easy obtainment of the target compounds in one step and with good yields.

**Figure 3:**
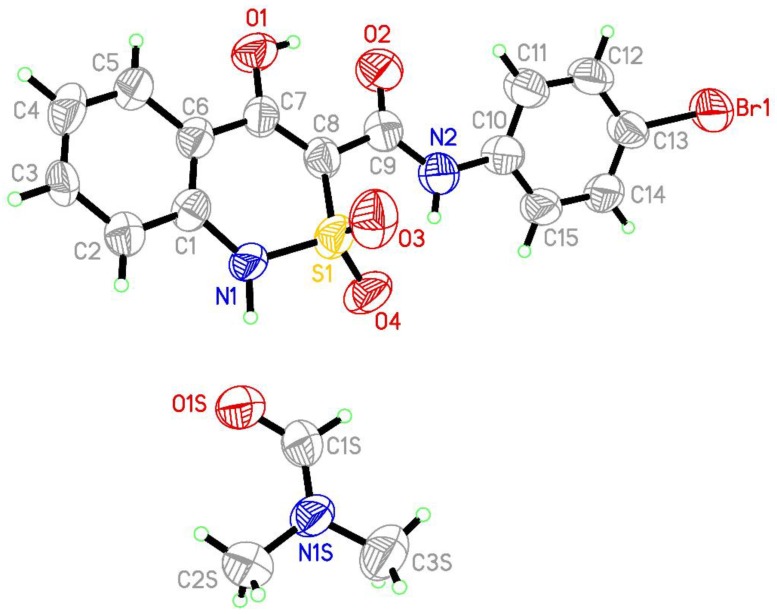
The standard representation of different atoms in different colors. The molecular structure of 4-bromoanilide **2l** *N*,*N-*dimethylformamide monosolvate with atoms represented by thermal vibration ellipsoids of 50% probability.

Most anilides **2а**–**l** are colorless crystalline substances, although sometimes some of them may be white samples with a slight yellowish tint. At room temperature they are moderately soluble, and when heated they are readily soluble in *N*,*N*-dimethylformamide (DMF) and dimethyl sulfoxide (DMSO). However, these solvents (at least DMF) should not be used for purification of anilides **2а**–**l**. Using X-ray diffraction analysis (Figure 3), the ability of the compounds studied to form stable solvates with DMF has been shown on the example of 4-bromo-substituted derivative **2l**. But the presence of DMF in any biologically active substance (taking into account its rather high toxicity) is exceptionally undesirable. Therefore, for the recrystallization of anilides **2а**–**l**, it is better to use other solvents—for example, easily removable methanol, methylene chloride, or their mixtures (see Materials and Methods).

In addition, according to the results of the X-ray diffraction study, interesting structural peculiarities have been revealed for 4-bromoanilide **2l**. The asymmetric part of the cell unit contains three molecules of this compound and three solvate molecules of DMF. At the same time, the dihydrothiazine heterocycle adopts a “twist-boat” conformation in all cases (the puckering parameters [30] are: S = 0.51, Θ = 41.0°, Ψ = 25.8° in molecule **А**, S = 0.58, Θ = 42.5°, Ψ = 26.2° in molecule **В**, and S = 0.56, Θ = 46.4°, Ψ = 24.5° in molecule **С**). Deviations of the S_(1)_ and С_(8)_ atoms from the mean plane of the remaining atoms of the cycle are 0.63 Å and 0.17 Å in molecule **А**, 0.73 Å and 0.21 Å in molecule **В**, and −0.74 Å and−0.22 Å in molecule **С**, respectively. The N_(1)_ atom has a pyramidal configuration (the sum of the valence angles centered on it is 355.6° in molecule **А**, 354.7° in molecule **В**, and 339.6° in molecule **С**).

The carboxamide fragment of the substituent at the С_(8)_ atom is coplanar to the С_(7)_–С_(8)_ endocyclic bond (the С_(7)_–С_(8)_–С_(9)_–О_(2)_ torsion angle is 3(1)° in **А**, 0(1)° in **В**, and 1(1)° in **С**). Formation of the О–Н···О hydrogen bond leads to the electron density redistribution in the О_(1)_–С_(7)_–С_(8)_–С_(9)_–О_(2)_ fragment. It is evident as elongation of the С_(9)_–О_(2)_ bond to 1.245(9) Å in **А**, 1.259(7) Å in **В**, 1.243(9) Å in **С**, and С_(7)_–С_(8)_ to 1.356(8) Å in **А**, 1.364(9) Å in **В**, and 1.366(8) Å in **С** compared to their mean values [31]: 1.210 and 1.326 Å, respectively. At the same time, the С_(7)_–О_(1)_ bond is shortened up to 1.332(8) Å in **А**, 1.349(8) Å in **В**, and 1.343(8) Å in **С** (the mean value is 1.362 Å). The *para*-bromophenyl cycle is located in the *ар*-position relatively to the С_(8)_–С_(9)_ bond and is turned relatively to the carboxamide fragment in molecule **А**, whereas in molecules **В** and **С**, it is practically coplanar to this plane (C_(8)_–C_(9)_–N_(2)_–C_(10)_ torsion angles are −169.8(8)° in **A**, −179.8(8)° in **B**, −171.9(8)° in **C**; C_(9)_–N_(2)_–C_(10)_–C_(11)_ 19(1)° in **A**, 4(1)° in **В**, and 8(1)° in **C**). In the crystal phase, the molecule 4-bromoanilide **2l** is bonded with the DMF solvate molecule by the N_(1)_–H···O_(1S)′_ intermolecular hydrogen bonds (H···O 1.73 Å, N–H···O 166° in **A**, H···O 1.75 Å, N–H···O 166° in **B**, and H···O 1.69 Å, N–H···O 166° in **C**).

According to the data of ^1^Н-nuclear magnetic resonance (NMR) spectroscopy, all 4-hydroxy-2,2-dioxo-1*H*-2λ^6^,1-benzothiazine-3-carboxanilides **2а**–**l** synthesized in DMSO-*d*_6_ solution also exist in 4-ОН form. This is confirmed by the singlets of the hydroxyl protons in the range of 15.5–15.0 ppm; they easily enter into deuterium exchange due to the pronounced acid properties of 4-ОН-groups and quickly disappear after adding D_2_O. A similar behavior is also observed in the singlet signals of protons of cyclic sulfamide groups, which originally resonate in a slightly stronger field—at 12.3–12.2 ppm. This indicates the acidic nature of these fragments, which cannot be said about acyclic anilide NH-groups—their protons appearing in the ^1^Н-NMR spectra as singlets at 10.0–9.4 ppm, on the contrary, enter into deuterium exchange very slowly. Analysis of the “aromatic” region of the proton spectra of anilides **2а**–**l** does not cause difficulties in general. However, sometimes it is difficult to make a specific assignment of one and all signals, since resonance frequencies of certain protons are very close or completely coincide. Complex multiplets observed in such cases can only be interpreted by the total integrated intensity.

The number and chemical shifts of signals recorded in the ^13^С-NMR spectra completely correspond to the structural formulas of the 4-hydroxy-2,2-dioxo-1*H*-2λ^6^,1-benzothiazine-3-carboxanilides **2а**–**l** studied. In the case of compounds **2b**–**e**, ^13^С-NMR spectroscopy allows us to reliably prove the presence of the fluorine atoms in their anilide fragments. This is shown by both significant paramagnetic shifts (more than 30 ppm compared to the unsubstituted analog **2а**) of the signals of carbon atoms associated with them and splitting of the atoms in the doublets with typical constants of the spin–spin interaction for C–F bonds in aromatic compounds (see Materials and Methods). In the ^13^С-NMR spectra of chloro-substituted anilides **2f**–**i**, there is also a shift in the weak field of signals of carbon atoms bound directly with halogens. However, it is expressed much less than in fluoroanilides **2b**–**e**. Due to the “heavy atom” effect, bromine affects the position of its neighboring carbon atom in the ^13^С-NMR spectra not so categorically: diamagnetic shielding (*ortho-*and *para-*bromoanilides **2j**, **2l**) is observed more often, a paramagnetic shift of the С-3′ signal in the spectrum of *meta-*bromoanilide **2k** is only 1.9 ppm.

**Figure 4:**
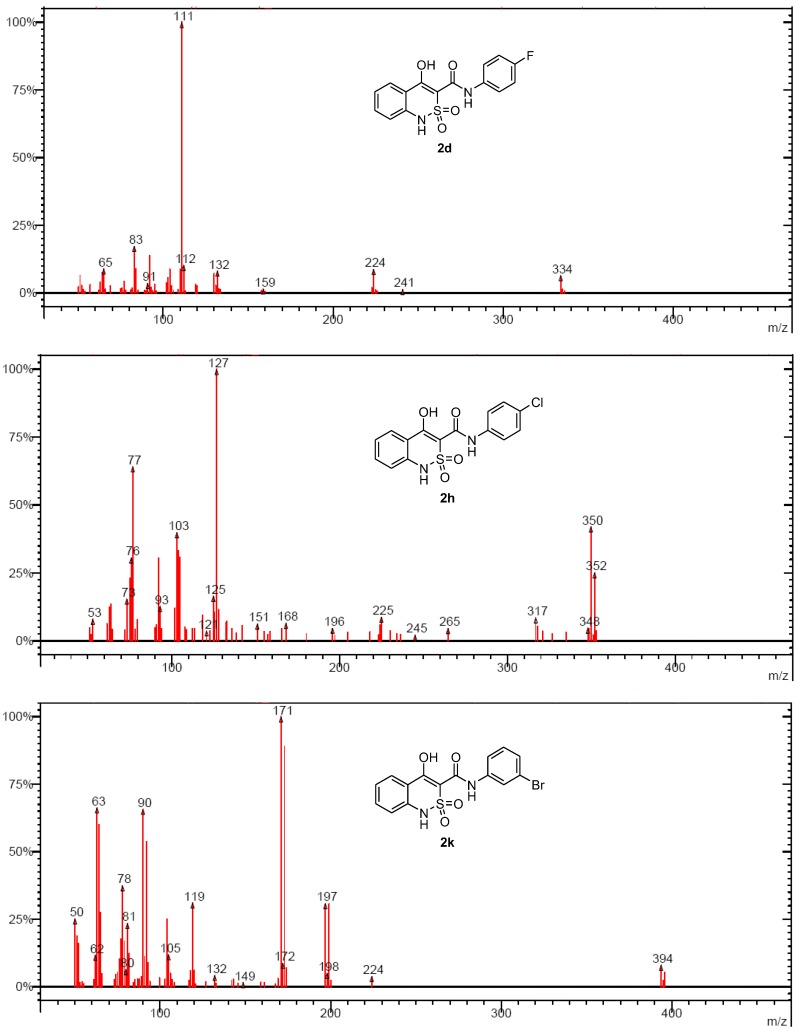
Examples of mass spectra of monohalogen-substituted 4-hydroxy-2,2-dioxo-1*H*-2λ^6^,1-benzothiazine-3-carboxanilides **2**.

Unlike fluorine, atoms of chlorine and bromine do not possess the magnetic moment and have no effect on the multiplicity of carbon signals related to them. For this reason, NMR spectroscopy is not able to reliably distinguish chloro- and bromo-substituted analogs. Mass spectrometry (MS) can solve such analytical problems easily. It was also used by us to confirm the structure of the substances synthesized. In the conditions of electron impact ionization, 4-hydroxy-2,2-dioxo-1*H*-2λ^6^,1-benzothiazine-3-carboxanilides **2а**–**l** do not have a high stability. As a result, the intensity recorded in the mass spectra of the peaks of their molecular cation-radicals usually is extremely low. However, in all cases these peaks are observed; and here, in addition to values of *m*/*z* (i.e., the molecular weight of each test sample) multiplicity is important. It gives additional useful information about the types of halogens that are present in the molecule and their number. For example, peaks of molecular ions of fluoro anilides **2b**–**e** are singlets regardless of the number of fluorine atoms, since fluorine is monoisotopic (Figure 4). In nature, chlorine exists as two isotopes: ^35^Cl and ^37^Cl in the ratio of 75.5 and 24.5%, respectively [32]. Therefore, in the mass spectra of monochloro-substituted anilides **2f**–**h**, the molecular ions appear in doublets with a more intense peak with a smaller *m*/*z* value, which corresponds to the ^35^Cl isotope (Figure 4). In one molecule, the dichloro-substituted anilide **2i** may contain isotopes of chlorine both with identical and different mass numbers. But according to their natural prevalence, the molecular ion peak in this case has the appearance of a triplet with the intensity ratio close to 9:6:1. A distinctive feature of the mass spectra of monobromanilides **2j**–**l** is the fact that both peaks in the doublet of their molecular ions are very close in intensity since bromine exists as two isotopes—^79^Br and ^81^Br in the ratio of 50.54 and 49.46%, respectively [32].

**Scheme 2:**
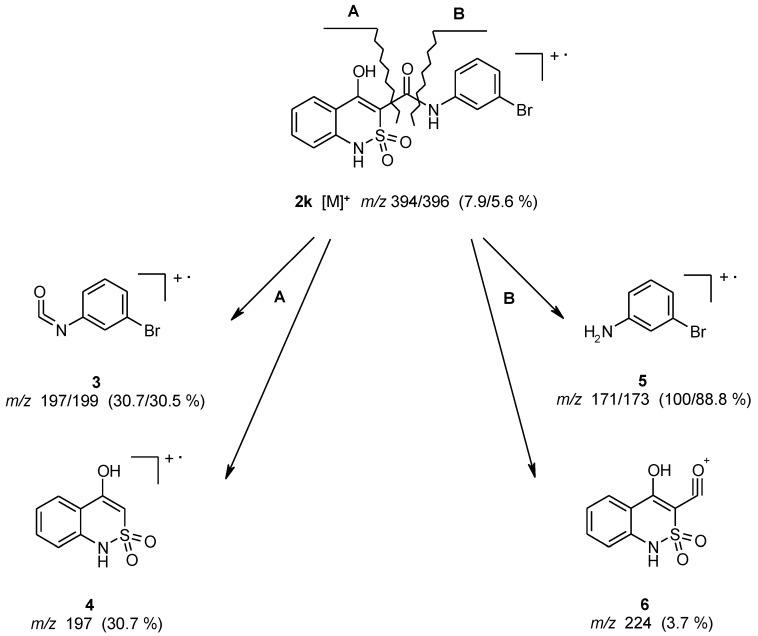
Primary fragmentation of the *N*-(3-bromophenyl)-4-hydroxy-2,2-dioxo-1*Н*-2λ^6^,1-benzothiazine-3-carboxamide (**2k**) molecular ion.

For hetarylamides [15,16,19,20], as well as alkyl- [22,25], hydroxy- and alkoxy-substituted [24] anilides of 1-R-4-hydroxy-2,2-dioxo-1*H*-2λ^6^,1-benzothiazine-3-carboxylic acids, two directions of the primary fragmentation of molecular ions are characteristic: breaking of the heterocycle–3-carbamide fragment bond (pathway А) or destruction of the acyclic carbamide bond (pathway В). It is interesting that in the case of anilide **2a** and its halogenated analogs **2b**–**l**, this behavior (Scheme 2) is recorded only in bromo-substituted derivatives **2j**–**l**. Obviously, peaks associated with the corresponding anilines are usually the most intense in the mass spectra of the compounds under study. It is noteworthy that fragment ions [M − Hal]^+^, being typical for halogen-containing compounds in the mass spectra of 4-hydroxy-2,2-dioxo-1*H*-2λ^6^,1-benzothiazine-3-carboxanilides **2b**–**l**, are observed only in the case of *ortho*-chloro-**2f** and *ortho*-bromo-**2j** substituted derivatives.

### 3.2. Evaluation of the Analgesic Activity

The results of studying the analgesic properties of all 4-hydroxy-2,2-dioxo-1*H*-2λ^6^,1-benzothiazine-3-carboxanilides **2а**–**l** synthesized are presented in Table 1. They show that in principle, halogenation of the anilide fragment can be an easy and very effective way of optimizing analgesics of the 2,1-benzothiazine series. A significant increase in the activity that accompanies the transition from the unsubstituted anilide **2а** to some of its halogenated analogs is evidenced in favor of this conclusion. Its positive role is also played by a virtually unlimited range of commercially available halogen-substituted anilines; it allows us to perform various chemical modifications of the base molecule without any special expenses. Thus, it is now clear that *ortho-*mono-substituted products are not of interest as analgesics irrespective of the halogen nature. At the same time, most of their *meta*- and *para*-isomers are able to suppress the pain response at the level of meloxicam. Sometimes the introduction of the second atom of halogen in the anilide part of the molecule may not substantially affect the analgesic effect, for example in the case of 3,4-difluoroanilide **2e**. However, 2,5-dichloroanilide **2i**, which significantly exceeds meloxicam by the level of specific activity, proves conclusively the feasibility of further research among halogen-substituted 4-hydroxy-2,2-dioxo-1*H*-2λ^6^,1-benzothiazine-3-carboxanilides with at least two halogen atoms in the anilide fragment.

## 4. Conclusions

This study presents halogen-substituted 4-hydroxy-2,2-dioxo-1*H*-2λ^6^,1-benzothiazine-3-carboxanilides synthesized as potential analgesics. Based on X-ray diffraction analysis, NMR spectroscopy (^1^Н and ^13^С), and mass spectrometry, it has been determined that in the crystalline form and in solution the substances obtained exist in the 4-hydroxy form. The ability of halogen-substituted 4-hydroxy-2,2-dioxo-1*H*-2λ^6^,1-benzothiazine-3-carboxanilides to form solvates with DMF has been experimentally confirmed, and it should be considered in their purification. According to the results of the pharmacological testing conducted, highly active analgesics have been found among the compounds studied. The structural and biological relationships identified are discussed. The expediency of further research among 4-hydroxy-2,2-dioxo-1*H*-2λ^6^,1-benzothiazine-3-carboxanilides with several atoms of halogens in the anilide fragment has been demonstrated.

## Figures and Tables

**Table 1:**
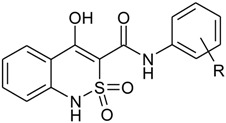
Analgesic Activity of Anilides **2a**–**l** and the Reference Drug.

Entry	Product	R	Latent Period in 1 h after Introduction of the Compounds, s ^a^	Change of the Latent Period, Compared to Control, %
1	**2a**	H	4.80 ± 0.11	+24.8
2	**2b**	2-F	3.89 ± 0.10	+1.1
3	**2c**	3-F	5.35 ± 0.13	+39.0
4	**2d**	4-F	5.77 ± 0.14	+49.9
5	**2e**	3,4-(F)_2_	5.66 ± 0.12	+47.1
6	**2f**	2-Cl	4.13 ± 0.13	+7.4
7	**2g**	3-Cl	5.02 ± 0.11	+30.3
8	**2h**	4-Cl	5.73 ± 0.14	+48.9
9	**2i**	2,5-(Cl)_2_	6.55 ± 0.16	+70.1
10	**2j**	2-Br	4.34 ± 0.10	+12.7
11	**2k**	3-Br	5.46 ± 0.14	+41.8
12	**2l**	4-Br	4.17 ± 0.11	+8.3
13	Meloxicam	–	5.71 ± 0.15	+48.2
14	Piroxicam	–	4.77 ± 0.13	+24.1
15	Control	–	3.85 ± 0.12	–

^a^ All results from biological tests were analyzed statistically using Student’s *t*-test. Effects were regarded as statistically significant at *p ≤* 0.05.
